# Supplementary material for: Are carbon emissions trading and green financial instruments synergistic? -Comprehensive quantitative research based on content analysis
Source: PLoS One. 2024 Mar 7;19(3):e0298601. doi: 10.1371/journal.pone.0298601 (PMC10919601; doi:10.1371/journal.pone.0298601)
Supplement: S1 Data — (ZIP) [file pone.0298601.s001.zip › Data/地级市Z_F工作报告.zip!/╜¡╬≈/╛┼╜¡╩╨/└┤╫╘╣1⁄2╓┌║┼í╛┬φ┐╦╩2╛▌═°í┐_2015.docx]

各位代表：

　　现在，我代表市人民政府，向大会报告工作，请予审议，并请各位市政协委员和列席会议的同志提出意见。

　　一、2014年工作回顾

　　过去的一年，在省委、省政府和市委的正确领导下，全市上下深入贯彻党的十八大和十八届三中、四中全会精神，认真学习贯彻习近平总书记系列重要讲话精神，扎实推进沿江开放开发和昌九一体化，有效克服经济下行的影响，经济社会发展保持了良好的势头，完成了年初确定的目标任务，迈出了“双核”发展的坚实步伐。全市完成生产总值1780亿元，同比增长10.3%。财政总收入328.5亿元，增长17.2%；公共财政预算收入213.7亿元，增长21.3%。固定资产投资1812.5亿元，增长20.2%。社会消费品零售总额496.4亿元，增长13.9%。居民消费价格总水平上涨2.1%。

　　（一）决战工业一万亿开局良好，工业主导地位更加凸显。围绕“三年翻一番，五年过万亿”的目标，全市上下聚焦大工业、决战大工业，工业主导型的经济格局更加凸显。规模以上工业主营业务收入净增近1000亿元，达到4731.1亿元；工业固定资产投资突破1000亿元，达到1249亿元；规模以上工业企业突破1000户，达到1121户；工业增加值945.5亿元，增长12.5%；利税总额480亿元，增长17%；工业用电量99.5亿度，增长13.8%。每季集中开工，压茬梯次推进，限期督查倒逼，全力推进工业项目建设，艾美特电器、攀森镍粒合金二期、亚东水泥六期等146个亿元以上项目竣工投产；九江石化油品质量升级、明阳线路板、吉恩重工海底电缆、春光线缆等116个亿元以上项目加快推进；神华九江电厂、兄弟医药、瑞智机电等216个亿元以上项目开工建设。帮扶企业提质增效、技改扩能、兼并重组，一批企业在盘活中做大做强，新增主营业务收入过亿元企业143户。优化产业布局，推进产业招商，完善产业链条，十大主导产业实现主营业务收入3950亿元，占全市比重82.5%；新能源、新材料、电子信息、先进装备制造、绿色食品等新兴产业实现主营业务收入1500亿元，增长20%；9个特色产业集群纳入省重点调度，永修有机硅、武宁节能灯成为省级工业示范产业集群。全市13个工业园区完成基础设施投入145亿元，新增开发面积55平方公里，所有园区主营业务收入过100亿元，8个过200亿元，九江经济技术开发区突破1000亿元；永修星火工业园获批国家新型工业化产业示范基地，成为江西省首个国家级出口工业产品质量安全示范区；德安县获批江西省纺织服装产业基地。

　　（二）城市建设统筹推进，门户形象加快提升。坚持老城与新区并建、规模与质量并重、建设与管理并举，促进城市建设转型升级，不断提高城镇化水平，城镇化率提高1.45个百分点，达到49.12%。《大九江都市区总体规划》编制有序推进，《九江城市总体规划》修编全面完成，瑞昌市、共青城等7个县（市）城市总体规划完成修编，城镇规划体系进一步完善。中心城区210个重点城建项目完成投资225亿元，长虹东大道、濂溪大道延伸线、八里湖北大道路桥工程、数字城管指挥中心等35个项目建成交付使用；九派诗廊、芳兰组团路网、濂溪片区截污工程、房产交易中心等37个项目基本建成；国际金融广场、绿地ICC大都会、通岭大道、兴城南大道、白水湖环湖公园、第四自来水厂、九江动物园搬迁等138个项目有序推进。投资5亿元的老城区八大类改造提升工程基本完成，整治改造边街小巷278条，改造提升主次道路13条，改造路面14.2万平方米、人行道3.2万平方米，改扩建停车场10个，新增停车泊位1938个，新建改造公厕43座，疏通改造排水管网2.3万米，新增路灯900余盏。全市完成棚户区改造拆迁2.9万户、307万平方米，其中中心城区1.4万户、154万平方米。“畅通九江”工程扎实推进，“城市之门”改造有序实施。全面开展城市环境综合整治“百日大会战”，脏、堵、乱、差、散、污等突出问题整治初见成效；市区新增新能源公交车100辆，市区至大千世界、星子县城至东林佛教文化游览区公交线路开通；大力创建国家森林城市，长虹大道、庐山大道、庐山南路等8条道路绿化升级改造全面完工，公园广场绿化、小游园绿地建设快速推进；德安、永修、星子、武宁、湖口、彭泽、九江县成功创建省级森林城市。城市水生态文明建设规划编制完成，开展PM2.5实时监测与发布，空气质量明显好转。县城建设亮点纷呈，各县共安排重点城建项目577个，完成投资380亿元；中心镇建设步伐加速，14个建制镇列入全国重点镇，城镇品位不断提升。

　　（三）昌九一体步伐加快，发展格局进一步优化。昌九联动有序推进，同城效应逐步显现。九江绕城高速、都九高速星子至九江段、昌九高速“四改八”通远段建成通车，昌九大道九江段建设快速推进；六大类25项合作事项有序推进，通信、户籍、金融、住房公积金贷款业务实现同城化，新农合直补、医疗检验检查结果互认和大气污染联防联控等工作进展顺利。沿江开放开发深入推进，带动效应不断增强。沿江四大板块完成基础设施投资133亿元，湖牛公路改造、500KV石钟山—洪源输变电等一批项目建设完工，九景衢铁路、武九客专、赛城湖大桥等一批项目加快推进；临港产业发展势头良好，实施亿元以上工业项目159个，总投资1573.5亿元，完成投资662.4亿元。沿江四大板块以全市三分之一的面积，拥有全市60%的规模以上工业企业、70%的规模以上工业主营业务收入、80%的50亿元以上项目，成为最具活力的增长区域。共青先导区建设有效推进，示范效应开始显现。220KV蒲塘输变电项目、南湖新区和工业园区60公里路网建成；实施亿元以上工业项目118个，泰然针织、龙泰运动器材产业园等54个项目建成投产。按照“权力下放、政策松绑、服务到位”的原则，加快县域经济发展升级，县级财政总收入完成262.6亿元，县县过10亿元，6个过20亿元，“沿江挑大梁、沿路做支撑、县区为依托”的发展格局基本形成。

　　（四）经济结构积极变化，第三产业不断壮大。旅游产业加快发展，出台了《关于深化改革创新加快旅游发展的意见》，编制完成了《大庐山旅游发展规划纲要》，组建了旅游发展委员会，成立了庐山旅游发展集团，打出了“庐山藏天下?九江通四海”品牌，旅游总人数7516万人次，增长33.9%；旅游总收入623亿元，增长55%。民生?大千世界梦幻乐园、瑞昌红木博览园等建成开放，庐山公路南、北线改造完成，庐山西海“一路三中心”基本建成，庐山索道、佛文化产业园、中国鄱湖水生态体验基地等项目加快推进。九江入选“最美中国旅游目的地城市”,全市新增4A级景区4个；庐山获评“最美旅游景区目的地”和“中国最美休闲胜地”；星子县入选全国旅游标准化示范县；武宁县被评为全国休闲农业与乡村旅游示范县。商贸物流稳步发展，新增物流企业151家，完成公路货运量1.14亿吨、货运周转量255.6亿吨公里，港口集装箱吞吐量22.4万标箱、货物吞吐量8000万吨；年交易额超20亿元的商品市场和商贸流通企业均达到4家，南方粮食交易市场获评江西省重点商品交易市场，长东物流园获评省重点物流园区；九方购物中心、21世纪家居中心开业，联盛九龙广场、联盛十里老街、喜盈门国际商业广场、禧徕乐国际商业中心等项目进展顺利。“万村千乡”市场工程扎实推进，3家配送中心、13家直营店、15个乡镇商贸中心建成。餐饮住宿、交通运输、邮政通信等传统服务业稳步发展；总部经济、楼宇经济和房地产业有序发展；家政服务、社会养老、中介服务等新兴服务业加快发展，恒盛科技园等电商产业园发展迅速，新增年交易额5000万元以上的电商企业4家，全年电子商务销售额150亿元。全市金融机构本外币存贷款余额1879亿元和1231亿元，分别比年初增加114亿元、164亿元。第三产业实现增加值638亿元，增长9%，占GDP比重提高0.7个百分点。

　　（五）“三农”工作持续加强，基础地位得到巩固。农业生产保持稳定，大宗农产品稳产增产，粮食生产实现十一连丰。农村土地流转面积132万亩，新建千亩以上特色农产品基地67个，新引进亿元以上农业项目32个，新增国家级农业龙头企业1家，创建现代农业示范园区25个。新增农民合作社775家、家庭农场814家。新增“三品一标”26个，“双井绿”“彭泽鲫”被核准为国家地理标志证明商标；“庐山云雾”“宁红金蕊”在2014年上海国际茶博会上获金奖。11个小农水重点县建设加快推进，新增恢复、改善灌溉面积27万亩，建设高标准农田10万亩，706处水毁水利工程修复任务全面完成，137座一般小（Ⅱ）型水库除险加固和30条中小河流治理项目主体工程基本完成。农村面貌有效改善，完成新农村建设点改水3万户、改路670公里、改厕3.4万户。新增农村安全饮水人口37.6万。完成县道升级60公里，新建农村公路1742公里、农村桥梁103座。63个重点镇、133个中心村加快建设，有序引导20万农民集中居住。城乡生态建设加快推进，造林绿化27万亩，“森林十创”和高速公路及环庐山沿线村庄提升工程基本完成，修河国家湿地公园通过国家验收。规范农民建房管理，“两违”现象得到有效遏制。严格落实各项强农惠农富农政策，加强农民技能培训，新增转移农村劳动力5.9万人，实施扶贫移民搬迁2.1万人，发放水库移民直补资金1亿元。农村居民人均可支配收入达到10139元，增长11.3%。

　　（六）改革开放力度加大，发展动力明显增强。全面深化改革，行政审批制度改革成效明显，取消市级行政审批及收费项目79项，审批时限压缩为36个工作日，减少前置中介服务事项70项，中介组织管理进一步规范。政府机构改革和职能转变顺利推进，农村集体土地承包经营权确权登记颁证和抵押贷款试点在全省率先开展，国有林场改革全面完成，财税、金融、注册资本登记、水利管理等改革稳步推进，长江河道采砂管理综合整治有效推进。对外开放水平不断提升，新批外商投资企业144家，实际利用外资14.5亿美元，增长17.8%；新引进市外5000万元以上开工项目377个，实际进资625亿元，增长9.2%；实现进出口总额57.7亿美元，增长21.8%，其中出口46.5亿美元，增长15.2%；九江海关纳入长江经济带区域通关一体化，九江口岸扩大开放和核心能力建设通过国家验收，检港联网系统正式运行，商品检验检疫实现通报通放，城西港区纳入全国启运港退税试点。科技创新能力不断增强，新增国家级工程技术研究中心5家、重点实验室1家，新增高新技术企业32家，新建院士工作站7家；九江船舶配套设备高新技术产业化基地通过国家审批，共青城市获批国家知识产权试点市。全市专利申请量2225件，授权量1137件，分别增长69.8%和29.2%。

　　（七）各项事业全面进步，人民生活有效改善。民生工程扎实推进，用于民生领域的财政支出292.5亿元，占公共财政预算支出的76.2%。新开工建设各类保障房7.3万套，基本建成8.2万套，其中中心城区新开工1.9万套、基本建成3.7万套，为1.9万户发放租赁补贴。新增城镇就业6.2万人，为城乡困难群众购买公益性岗位5000个，城镇居民可支配收入达25077元，增长10.2%。低保、五保和精简退职困难老职工提标提补惠及27.6万人，城乡医疗救助覆盖30万城乡困难群众，实施重大病医疗救助17.3万人次，中心城区“三无”人员分类保障水平不断提高。新建城郊蔬菜基地6000多亩，中心城区新建改造农贸市场4家，建立惠民平价蔬菜直销区20个，放心粮油工程15家配送中心、110家示范经营点建成运营。基层政权建设和民主管理进一步加强，社区办公条件有效改善，社区干部待遇逐步提高，浔阳区荣获“全国和谐社区建设示范区”称号。社会事业全面发展，全市财政教育投入70.5亿元，新改扩建校舍面积38万平方米，补充教师1700名；湖滨片区教育网点优化布局，九江职大新校区、九江小学八里湖校区交付使用，中心城区4所省定标准幼儿园建设顺利推进。医疗卫生服务体系不断完善，新农合参合率达98.4%，“先看病、后付费”模式惠及参合农民61万人，免费救治八种重大疾病病人1.2万名；市三医院内科住院大楼、市五医院综合住院大楼投入使用，大病医疗补充保险实现全覆盖。公共文化服务体系建设扎实推进，市文化艺术中心、琵琶亭生态文化园建成使用，市县文化四馆、乡镇综合文化站、村农家书屋等公共文化设施实行免费开放。体育事业快速发展，联盛足球队成功跻身中国足球甲级联赛，九江籍运动员在仁川亚残会上获得两块金牌。农产品质量监管进一步加强，食品药品安全检测能力明显提升。“单独两孩”政策稳步实施，人口自然增长率6.96‰。城乡建设用地增减挂、低丘缓坡荒滩等未用地、工矿废弃地复垦试点工作有序推进。气象防灾减灾能力进一步加强。严格环保准入，强化环境监管，扎实开展环保专项行动，省政府下达的节能减排任务全面完成。安全生产形势平稳，事故起数和死亡人数实现双下降。信访形势总体稳定，应急处置能力进一步提升，市政府民声直通车受理和办理群众意见、建议、诉求12338件，办结率96.7%。扎实推进“天网”工程四期、“封城计划”二期建设，深入开展治安、禁毒等专项行动，公众安全感明显提高；公安队伍建设不断加强，涌现出全国公安系统二级英模柯善梅等先进人物。统计、审计、水文、盐业、通信、无线电管理、档案、司法、国安、保密、编制、地方志、对台、外事侨务、民族宗教、驻外联络等工作得到新加强；国防动员、民兵预备役、双拥创建、人民防空、防震减灾、消防等工作取得新成绩；妇女儿童、老龄、残疾人、志愿者、红十字和慈善等事业实现新发展。

　　按照中央要求和省委、市委部署，市政府及其部门扎实开展党的群众路线教育实践活动，“四风”问题得到有效治理，政府自身建设进一步加强。严格按照法定权限和程序开展工作，自觉接受市人大及其常委会的法律监督、工作监督；积极支持政协履行职能，推进协商民主政治建设，认真听取各民主党派、工商联和无党派人士意见，办理市人大代表建议253件、市政协提案461件。认真落实中央“八项规定”和国务院“约法三章”精神，“三公”经费支出下降27.4%。廉政建设力度加大，深入开展了“红包”治理等专项整治，严肃查处了一批损害群众利益的突出问题，坚决查办了一批违纪违法腐败案件。

　　各位代表，这些成绩的取得，是省委、省政府高度重视、高位推动和市委正确领导的结果，是市人大、市政协有效监督、全力支持的结果，是全市人民团结一心、共同奋斗的结果。在此，我代表市人民政府，向全市广大干部群众，向各位人大代表、政协委员、各民主党派、工商联、无党派和社会各界人士，向驻浔单位、人民解放军、武警官兵、公安民警和离退休老同志，向所有关心、支持、参与九江建设发展的朋友们，表示衷心的感谢并致以崇高的敬意！

　　在总结成绩的同时，我们也清醒地看到：经济总量不大、综合实力不强，仍是当前的主要矛盾，做大九江、打造“双核”的任务繁重；产业结构不尽合理，产业层次偏低，创新能力不强，加快发展升级仍然任重道远；经济运行下行压力进一步加大，企业生产经营困难增多，决战工业一万亿任务艰巨；促进城乡居民持续增收难度较大，影响社会和谐稳定的因素依然存在；行政效能有待进一步提高，发展环境有待进一步优化。对此，我们将采取有力措施，认真加以解决。

　　二、2015年工作安排

　　2015年是全面深化改革的关键之年，是全面推进依法治国的开局之年，也是全面完成“十二五”规划的收官之年。尽管世界经济仍处在国际金融危机后的深度调整期，但我国仍处于可以大有作为的重要战略机遇期，特别是国家推进长江经济带建设，做大九江，我们机遇更多；尽管区域竞争依然激烈，但省委、省政府把九江作为全省“双核”之一来打造，上级的支持力和外界的注意力正在加速转化为发展力，做大九江，我们信心更足；尽管发展过程中依然存在困难和压力，但在市委的坚强领导下，全市上下已形成浓厚的发展氛围，凝成强大的工作合力，做大九江，我们基础更好。我们必须牢牢把握新机遇，积极挖掘新动力，努力形成新优势，全力做好今年经济社会发展的各项工作。

　　今年政府工作的总体要求是：深入贯彻党的十八大和十八届三中、四中全会精神，认真学习贯彻习近平总书记系列重要讲话精神，全面落实省委、省政府和市委的决策部署，主动适应经济发展新常态，围绕做大九江的目标，保持战略定力，深化改革创新，建设生态文明，加强民生保障，推进工业总量翻番、门户形象提升、旅游转型升级，奋力开创“双核”发展新征程。

　　全市经济社会发展主要预期目标是：生产总值增长10%；财政总收入增长15%；固定资产投资增长18%；社会消费品零售总额增长13%；居民消费价格总水平涨幅控制在3%；城镇居民人均可支配收入增长10.5%，农村居民人均可支配收入增长11%；人口自然增长率控制在8‰以内；完成节能减排任务。

　　为实现上述目标，重点抓好七个方面的工作：

　　（一）着力放大九江的独特优势，加快融入长江经济带

　　依托黄金水道建设长江经济带，是国家着眼区域发展全局作出的重大战略决策。九江作为长江中游的关键节点和重要支点，是江西融入长江经济带的主阵地、主平台和主抓手，必须加强研究、主动对接、积极作为，充分放大黄金水道优势、区域比较优势和独特区位优势，在全面融入中争取更多政策、拓展更大空间、实现更快发展。当前，重点要在做优发展平台上下工夫。

　　做优交通平台。交通是经济发展的“大动脉”，立体交通是做大九江的重点依托和重要前提。铁路方面：加快推进九景衢铁路、武九客专及长江大桥改造等项目建设，做好合安九、昌九高铁和常岳九铁路等项目前期工作，逐步实现与京沪、沪昆、昌吉赣高铁的有效对接，加快融入“北、上、广”四小时高铁网。公路方面：大力推进昌九大道、昌九高速全线“四改八”、都九高速、修平高速等项目建设，积极协调彭泽至东至高速公路安徽段建设，逐步打通昌九毗邻地区的断头路。机场方面：全面实施九江机场跑道加厚和通信导航升级工程，启动机场与昌九高速连接线建设。通过构建立体交通网络，逐步形成多式联运的大交通格局。

　　做优港口平台。以港强工，以工兴城，港城一体，依托九江152公里长江黄金岸线，努力把九江港打造成为“立足九江、服务全省、辐射周边”的区域枢纽港、长江喂给港。优化港口布局，在做强城西港区的基础上，重点推进彭湖板块红光综合枢纽港建设，加快实施中心城区散杂货码头整体搬迁，着力形成城西以集装箱为主、红光以散杂件为主的港口功能布局。构建集疏运体系，加快推进港口铁路专用线、疏港通道、公共锚地和航道建设。完善服务配套，集约利用岸线资源，严格控制业主码头，大力整治非法码头；积极引进港口、航运等企业，扶持本土船运企业发展；完善运输、仓储、配送等物流链条，加快形成以港口为枢纽的物流网络。

　　做优口岸平台。口岸是对外开放的门户。提高通关效率，推进电子口岸和昌九口岸一体化信息平台建设，实行关检合作“一次申报、一次查验、一次放行”的通关模式。拓宽通关领域，加大综合保税区申报力度，推进进口粮食、木材等专项产品指定口岸建设。密切通关协作，加强九江港与上海外高桥码头、洋山港的通关协作，打通南昌港至九江港绿色通道，积极融入长江流域通关一体化。

　　做优腹地平台。腹地是沿江开放开发的重要支撑和纵深空间。按照“找准支柱、做强支点、夯实支撑、优化支持”的要求，进一步完善考评体系，优化发展环境，支持引导县域发挥各自优势，夯实基础条件，壮大支柱产业，实现升级发展，拓展市域腹地。按照“规划一体化、基础设施一体化、公共服务一体化和产业互补对接”的总方向，推进昌九一体，抓好昌九合作事项的推进实施，加快共青先导区建设，拓展省域腹地。开展跨江合作，鼓励沿江县区先行先试，加快与宿松、望江、黄梅、武穴等地联动发展，提升“一江两岸”协作水平；积极参与长江流域跨区合作，主动对接上海自贸区，承接长三角产业转移，拓展流域腹地。

　　（二）坚定不移决战大工业，确保实现三年翻番

　　坚持引进增量、盘活存量、提升质量相结合，全力做大工业经济总量，力争实现规模以上工业增加值1100亿元、利税总额550亿元、主营业务收入6000亿元。

　　加快产业转型升级。产业转型升级是做强工业的必由之路。做大龙头企业促升级，推进九江石化油品质量升级改造、星火有机硅扩改一体化等项目建设，促进理文化工、赛得利化纤、江铜铅锌冶炼、攀森新材料、九江电厂等企业做大做强，吸引集聚一批产业链项目，形成龙头企业顶天立地、配套企业铺天盖地的发展格局，力争石油化工、现代轻纺产业主营业务收入突破1000亿元，钢铁有色产业达到700亿元、电力新能源产业达到500亿元。建设重大项目促升级，推进总投资350亿元的汉能光伏、300亿元的神华九江电厂、130亿元的北汽重组昌河汽车、67亿元的港森联源新材料、60亿元的五星纸业、50亿元的兄弟医药、50亿元的荣侨电子、50亿元的九宏新材料、50亿元的西矿铜业等重大项目建设，尽快实现达产达标，成长为主业突出、竞争力强的产业新龙头，形成一批产能规模达“千万级”“百万级”“十万级”的优势产能基地。提高创新能力促升级，发挥企业主体作用，加强与大专院校、科研院所的对接合作，引进培养一批科技领军人才和创新团队，推进院士工作站建设，利用本地高校优势，引导创新要素向企业集聚，支持企业研发、承接和采用新技术，培育引进一批高新技术企业，建设一批省级科技园区、基地和孵化器，全面提升产品和产业层次。

　　促进企业提质增效。企业提质增效是做强工业的当务之急。达产达标提质增效，强化项目要素保障和企业帮扶措施，促进在建项目尽早竣工投产，尽快形成产能、发挥效益，促进现有企业开足马力，全面达产达标、提高效益。技改扩能提质增效，加快现有企业生产工艺、装备升级换代，促进企业产品升级、质量提升，提高企业规模化生产水平和市场竞争力。整合重组提质增效，引导中小企业走靠大联强的路子，支持有条件的企业通过资产收购、产权受让、合资合作等方式联合重组，盘活企业存量资产和有效资源。节能减排提质增效，大力推广先进节能减排工艺和设备应用，抓好重点行业、重点企业、重点领域节能减排和重点工程建设，提升能效水平。力争全年新增规模以上工业企业100户以上，新增主营业务收入50亿元以上企业2—3户、10亿元以上5—8户、亿元以上80户。

　　优化园区功能配套。优化园区功能配套是做强工业的务实之举。进一步完善生产、生活、生根和生态功能配套，加快产城融合，提升园区承载力。完善园区水、电、路、气等管网和防洪排涝、污水处理设施，加快推进220千伏瑞昌码头输变电、湖口液化天然气储配和九江金鸡坡油库扩建工程，积极配合特高压入赣工程建设。以社会化综合服务体系建设为重点，加快园区公租房、学校、商业场所等公共服务设施建设，完善园区餐饮住宿、子女上学、就医、购物、休闲等生活服务功能。清理园区闲置用地和空置厂房，严格执行园区用地投资强度、容积率等控制标准，推进工业地产和多层标准厂房建设，提升园区用地集约化水平，实现企业在园区生根发展。做好园区生态建设规划，坚持生态建设、绿化美化与项目建设同步进行，加快建设生态园区。

　　推进服务优质高效。应对经济下行压力加大、企业发展困难增多，更加需要综合施策，打好组合拳，积极帮助企业解决生产经营中的困难，为项目建设、企业运行、产业发展创造良好的投资环境。按照“六个一”的工作机制，继续开展市领导联系千亿产业、挂点帮扶百亿企业工作，完善挂点联系会商通报制度。开展“百个部门帮百企”和“百个企业评部门”活动，强化客商投诉中心建设，有诉必应，一问到底。建立产业发展引导基金，完善企业上市激励政策。加强政银企对接，放大财园信贷通、助保贷等融资方式的撬动作用，破解融资难题。推进校企对接，加强技能培训，拓宽招工渠道，破解用工难题。

　　（三）加快推进新型城镇化，着力打造门户城市

　　牢牢把握新型城镇化转型升级的新要求，进一步完善城市功能，加强城市管理，提升城市形象，推进新型城镇化健康发展，逐步把九江打造成为省域门户城市和长江经济带重要节点城市。

　　坚持规划引领，加快构建大九江都市区。编制完成大九江都市区总体规划和专项规划，积极推进“多规合一”，加快构建以中心城区为核心、中等城市为节点、县城为支撑、集镇为基础的新型城镇化发展格局。着力提升新城区、改造老城区、整合拓展区、构建都市区，加快九江县撤县设区步伐，推进赛城湖片区与八里湖新区对接融合，逐步实现芳兰、威家、海会、赛阳片区组团发展。加强对县城及集镇的分类指导，鼓励支持共德永、瑞昌等向中等城市发展，积极扶持其他县城发展成为特色鲜明、宜居宜业的小城市，培育壮大一批沿江特色产业集镇、沿路物流节点集镇、滨湖生态旅游集镇、山区边贸中心集镇。

　　完善功能配套，增强中心城区承载力。加快完善服务功能。推进八里湖中央商务区、国际金融广场、市中心幼儿园八里湖分园、九江职业技术学院新校区、第四自来水厂、九江动物园等重大项目建设；加快改造三马路、考棚路、啤酒厂等片区棚户区，改造面积200万平方米；按照“水通、路平、灯亮、线齐、墙美”的标准，再投入5亿元，重点改造提升一批小巷道路、人行道、排水沟、路灯等破烂、破旧、破损设施，新建改造一批农贸市场、社区用房、公共停车场、公交场站等便民服务设施。加快完善交通功能。继续实施畅通工程，完成通岭大道、兴城南大道、琴湖大道（南段）等项目建设，完善提升环庐山公路，推进城区和环庐山公交化改造；启动火车站广场改造和重要结点立体交通设施规划建设；完善主次干道人行道、红绿灯、指示牌等设施，加强城市公交、出租车管理，市区再新增100辆新能源公交车；继续实施“城市之门”工程，协调推进绕城高速收费站“拆四建二”外移工程。加快完善生态功能。再投入2亿元用于森林城市创建，继续实施好城市绿化工程，建成濂溪文化生态公园、浔南城市森林公园、九龙山森林公园，建设八里湖等城市绿道系统，新建一批城市小游园，把九江打造成大树成荫、小树成林、鸟语花香、景色宜人的生态城市；推进城市水生态文明建设，加快城区易涝地段排涝设施改造，完成龙开河、濂溪片区截污管网改造，加强八里湖、赛城湖等中心城区湖泊水环境保护。加快完善文化功能。丰富街区、公园、建筑、雕塑等城市元素文化内涵，发挥公共文化设施的作用，提高市民文化素养和城市文明程度。

　　加强城市管理，提升城市品位。深入开展中心城区城市环境综合整治，巩固“百日大会战”成果，健全长效机制，打造干干净净的市容环境、整整齐齐的城市容貌、漂漂亮亮的城市景观、规规矩矩的公共环境、平平安安的出行环境、宜居宜憩的人居环境，力促中心城区面貌发生根本改变。加强城管综合执法、交警、环卫、志愿者等队伍建设，进一步完善管理职能、理顺管理体制、提高管理水平。积极引导社会资本进入环卫、市政、园林、物业管理等领域，做好已建公建项目的验收、移交和监管工作。坚持政府主导、市场运作，推进“智慧城市”建设，促进信息化与产业发展、社会管理、公共服务相融合，提高城市管理服务的数字化、精细化、集成化水平，为市民群众提供便利化、个性化服务。

　　（四）稳粮增收调结构，扎实做好“三农”工作

　　坚持在决战大工业中发展农业，在打造大门户中繁荣农村，在改善大民生中富裕农民，继续夯实农业稳定发展的基础，着力稳住农村持续向好的局势。

　　推进农业升级。坚持走产出高效、产品安全、资源节约、环境友好的现代农业发展道路，以稳定粮食生产为首要任务，稳步推进大宗农作物改种、改水、改杂，进一步提升传统农业规模化、机械化、产业化水平。大力培育新型经营主体，探索建立农村集体资产资源交易平台，引导促进农地、林地、水面等有序流转。加快农业项目建设，新增规模以上农业企业20家，新建千亩以上农产品基地70个，争创一批国家级和省级现代农业科技示范园，巩固城郊蔬菜基地建设成果。推进农产品品牌创建，打响鄱湖水产、庐山云雾茶、宁红工夫茶等特色品牌，发展“三品一标”等农产品，争创省级以上农产品品牌70个。强化农产品质量安全监管，加强标准化基地和农产品追溯体系建设，新增专业化统防统治服务面积10万亩。

　　加快农村发展。夯实农村发展基础，推进高标准农田建设，加快完善末级渠系，解决农田灌溉“最后一公里”问题，新增恢复灌溉面积7.8万亩，改善灌溉面积28.7万亩。完成1645座（处）水毁水利工程设施修复，解决25万农村人口饮水安全问题。加强农村公路管护，新建农村公路1000公里。改善农村人居环境，完善重点乡镇规划及中心村庄规划，加强农民建房管理，推进19个重点乡镇、38个中心村镇村联动建设，抓好831个新农村建设点村庄整治和垃圾无害化处理。推进农村生态建设，全面完成林地（湿地）保有任务；实施好国家农发水保项目，完成综合防治水土流失面积33.8万亩。

　　促进农民增收。认真落实强农惠农政策，充分挖掘农业内部增收潜力。发放“财政惠农信贷通”贷款5亿元以上，发展农民合作社350家、家庭农场350家、规模种养大户500户。加强农民培训和转移，强化就业服务，维护农民工合法权益，新增转移农村劳动力5.5万人。培育新型职业农民3000人，完成“一村一名大学生”工程招生600人，完成深圳技师学院“九江扶贫班”招生100人。扎实推进395个贫困村的村庄整治，继续完成2万人的扶贫移民搬迁任务，落实好水库移民现金直补和后期扶持项目的实施。

　　（五）发展文化旅游和商贸物流，繁荣现代服务业

　　繁荣现代服务业，既有利于优化经济结构、推动产业升级，又有利于优化发展环境、提升对外形象。文化旅游、商贸物流，是九江发展现代服务业的主要方向。

　　做强文化旅游业。围绕“五个一”的目标，编制完成旅游产业创新发展规划、旅游重点产业集群发展规划。放大庐山品牌优势，加快整合旅游资源，构筑以庐山为龙头的山上山下联动发展、城里城外相向发展的大格局。加强旅游资源开发，加快引进落户一批重大文化旅游项目，重点推进庐山索道、庐山建成区改造升级、归宗景区、庐山西海国际养生度假区、修水黄龙山旅游区综合开发等项目建设。加大旅游对外宣传力度，打响“庐山藏天下?九江通四海”品牌。抓好景区创建，支持庐山创建“国家旅游发展综合示范区”、庐山西海创建国家5A级景区、武宁创建“全国最美县城”。加快促进文化与旅游融合发展，充分挖掘九江丰富的历史文化，支持发展文化旅游产业。发展智慧旅游，推行标准化行业管理，加强旅游服务人才培养、商品开发和市场监管，支持旅游企业做大做强。力争全年旅游接待总人数、旅游总收入增长20%以上。

　　做旺商贸物流业。重点加大对省内货物向九江港集散的支持引导力度，增加南昌—九江、九江—上海洋山港航班，支持陆路物流枢纽港建设。拓展物流服务线路，培育发展第三方物流企业，提升物流配送服务水平。推进万达广场、九江新天地、九江茶市等项目建成，加快喜盈门国际商业广场、赣北果品批发市场、天马建材城升级改造、京九仓储物流园、赣电物流园等项目建设。依托“万村千乡”市场工程，推进乡镇商贸中心建设，打造一批省级商贸强镇，完善农村流通网络，健全城乡流通体系。

　　做优新兴服务业。注重发挥金融业在促进服务业加快发展中的作用，加快引进北京银行等金融机构入驻九江，支持发展村镇银行、农村资金互助社、融资性担保机构等新型农村金融机构。制定电子商务发展专项规划，加快建设电子商务示范园区，重点推进共青城电商物流产业园、中国邮政（九江）电子商务产业园和联盛易佳电子商务产业园建设，完善电子商务物流支撑体系，力争年交易额过5000万元电商企业达到15家。积极发展文化创意产业，重点打造数字出版、动漫游戏、新媒体等产业链，推动文化创意产业与相关产业融合发展。积极引导和鼓励社会力量发展养老产业，大力促进研发设计、商务会展、服务外包、教育培训、医疗保健、体育健身休闲、家庭服务等新兴服务业发展。

　　（六）推进改革开放，进一步增强发展动力

　　改革为加快发展提供动力，开放为做大九江注入活力。坚定不移实施大开放、推进大改革,在扩大开放中赢得发展机遇，在深化改革中拓宽发展空间。

　　推进重点领域改革。顺应时代要求，适应发展需要，坚持问题导向，加快重点领域和关键环节改革。继续深化行政审批制度改革，坚持简政放权、放管结合，在承接好上级下放审批事项的同时，再取消和下放一批市级审批事项，全面清理非行政许可审批，大幅度减少前置审批；深化政府机构改革和职能转变，完成事业单位分类申报与岗位设置管理工作，实施政府购买服务试点；加快行政机关及参公事业单位公务用车制度改革；深化财税体制改革，全面推行“营改增”；贯彻实施新预算法，实行预决算公开、审计公开和财政预算绩效管理制度，加强政府性债务管理；按照国家政策，推进机关事业单位养老保险制度改革，完善工资制度，提高基本工资标准并建立正常调整机制；推进新型农村社会养老保险和城镇居民社会养老保险并轨，逐步建立统一的城乡居民基本养老保险制度；深化户籍制度改革，完善教育、医疗、保障性住房等配套政策；深化农村综合配套改革，全面完成农村集体土地承包经营权确权登记颁证，积极开展农村集体经济组织产权制度改革试点，稳妥推进不动产统一登记和征地制度改革，探索建立城乡统一的建设用地市场。

　　扩大对内对外开放。大招商带来大发展，大开放促进大崛起。牢固树立“让大利、招大商、大发展”的理念，推动招商引资朝着优化结构、拓展深度、提高效益方向转变，确保全市实际利用外资16亿美元，引进市外5000万元以上项目资金1000亿元。强化产业招商，围绕重点产业，加强与国内、国际500强企业的对接，盯紧关联度大、带动性强、科技含量高的重大项目，加强跟踪对接，实行“一企一策”，确保企业落户。强化重点招商，积极组团参加国家和省举办的各类招商活动，重点开展好赴香港、澳门、台湾、江浙沪深等地区的招商，提高招商引资的针对性和实效性。强化商会招商，密切与各行业商会（协会）的联系，从招企业家到招商会转变、从专业招商到综合招商转变。加大筑巢引凤力度，加强感情联络、乡情吸引，支持本土人才大胆创业、在外浔商回归创业、务工人员返乡创业，掀起全民创业的新热潮。加快出口商品质量示范区建设，支持企业进口国际先进生产设备、关键技术和重要原材料，完成进出口总额59亿美元。

　　（七）倾力保障改善民生，促进社会和谐稳定

　　像抓经济建设一样抓民生保障，像落实发展指标一样落实民生任务，力求让发展成果更多、更公平地惠及全体人民，努力提高人民群众的生活质量和幸福指数。

　　做实民生工程。加大民生投入，全面完成省政府下达的民生任务。加大就业创业扶持力度，确保新增城镇就业5万人，为城乡困难群众购买公益性就业岗位4400个以上，培训农民工3.3万人，积极扶持大学生创业就业。新建公共租赁住房1.4万套，改造城市、工矿、林区、垦区等各类棚户区2.2万户，基本建成保障房5.1万套。健全保障性住房后期管理机制，盘活保障房存量资源。巩固新型农村合作医疗制度，新农合人均筹资标准提高到450元，各级财政补助提高到360元。探索建立特困家庭大病救助机制，加大孤儿和困境儿童保障力度。推进殡葬改革，启动市殡仪馆改造。加大扶贫开发和济困帮扶力度，面向特定人口、具体人口，实现精准脱贫，防止以平均数掩盖大多数。

　　发展社会事业。坚持教育优先，深化教育领域综合改革，制定实施第二轮学前教育三年行动计划；加快农村义务教育学校标准化建设和薄弱学校改造，推进农村教师周转宿舍建设试点，加强农村义务教育阶段师资队伍建设；努力办好特殊教育，支持发展民办教育，大力发展职业教育，加快共青科教城建设。实施文化亲民工程，推进基层公共文化标准化、均等化建设，提升公共文化场馆综合服务能力，争创国家公共文化服务体系示范区。完成边远乡村7万户广播电视直播卫星户户通工程。继续深化医药卫生体制改革，稳妥推进基层医疗卫生机构和县级公立医院改革，促进基本公共卫生服务均等化；推进鄱阳湖血吸虫病传染源控制策略推广区建设，继续推进八项重大疾病免费救治，抓好重大疾病防控工作。稳定低生育水平，提高出生人口素质，扎实推进“人口信息化”工程。健全食品药品安全监管体系，加强专项整治，保障公众饮食用药安全。开展全民健身运动，发展竞技体育，推进体育产业发展。开展第二次全国地名普查。加强基层政权建设和民主管理，完成第九届村（居）委会换届选举。强化防灾减灾功能，开展综合减灾示范社区创建，加快气象灾害移动指挥系统建设。深化全民国防教育，加强国防后备力量建设，争创“双拥模范城”全国五连冠、全省七连冠，巩固和发展军政军民团结，推动军民融合深度发展。启动编制“十三五”规划。充分发挥统计、审计、物价、工商、消防、盐业、人民防空、民族宗教、外事侨务、涉台事务、驻外联络、新闻出版、档案、地方志、保密、编制、无线电管理、妇女儿童、老龄、残疾人、志愿者、红十字和慈善等工作在促进发展、保障民生、构建和谐中的积极作用。

　　加强社会治理。健全重大决策社会稳定风险评估机制和应急管理机制，提高突发公共事件防范处置能力。加强信访工作，高度重视网上信访，开展“大接访”活动，畅通诉求渠道，实行诉访分离，有效解决群众合理诉求。贯彻落实新《安全生产法》，严格安全生产责任制，深入开展隐患排查治理，建立健全事故隐患排查治理体系和重大事故隐患挂牌督办机制，坚决遏制重特大安全事故的发生。推进法治九江建设，制定出台《法治九江建设纲要》，开展“六五”普法验收。加强医患等重点领域和行业人民调解组织建设。继续实施“天网”工程、“封城计划”，健全社会治安防控体系，强化公共安全管理，严厉打击各种违法犯罪，切实增强公众安全感，全力维护国家安全。

　　强化环境保护。严格执行《环境保护法》，加快生态文明先行示范区建设，保护好九江的青山绿水，努力让天更蓝、地更绿、水更净、空气更清新。坚持源头严防，加强修河等重要饮用水水源地保护，加强长江河道和鄱阳湖采砂统一管理；强化土壤污染源头综合整治，抓好土壤重金属污染和矿山治理修复；加强森林资源保护管理，提升森林质量和生态功能。坚持过程严管，健全环境安全应急处理体系和重点污染源在线监控体系，加快城镇污水收集管网和工业园区污水处理设施建设，加强环境敏感区的生态保护；继续实施PM2.5实时监测与发布，加大工业污染、农业面源污染和城乡生活污染减排与治理力度，完成减排任务。坚持后果严惩，加强环境监管执法，继续开展环保专项整治行动，严厉查处各类环境违法违纪行为。倡导生态理念，大力发展绿色低碳经济，抓好资源节约集约利用，推进生态与经济协调发展。

　　三、加强政府自身建设

　　全面依法治国，对政府自身建设提出了新的更高要求。强化宪法意识和法治观念，加快转变政府职能，规范行政行为，提高行政能力，建设法治政府。

　　（一）依法完善决策机制。按照公众参与、专家论证、风险评估、合法性审查、集体讨论决定的法定程序，规范重大行政决策行为。积极探索和引进第三方评估模式，建立决策后评估和纠错制度。推行政府法律顾问制度，健全重大行政决策合法性审查制度，强化重大决策终身责任追究制度及责任倒查机制，推进决策的科学化、民主化、法制化。自觉接受市人大及其常委会法律监督和政协民主监督，主动接受司法监督、党内监督和行政监察，虚心接受社会舆论、新闻媒体和群众监督。认真办理人大代表建议议案和政协提案。

　　（二）依法高效履行职责。按照“职权法定、权责一致”原则和“法定职责必须为、法无授权不可为”要求，推行政府权力清单、责任清单和负面清单管理，落实行政纠错问责制，依法规范行政行为。全面推行重点领域和关键环节的信息公开，推进行政服务标准化和网上审批系统建设，确保行政审批事项全部入网运行，提高行政服务效率。加强中介组织清理、整顿和监管，推进中介服务机构与主管部门、挂靠单位彻底脱钩。坚持勤勉理政，建立主体明确、层级清晰、具体量化的岗责体系，明确目标进度，强化过程监管，依法高效抓好落实。

　　（三）依法规范执法行为。加快推进行政执法体制改革，整合行政执法力量，逐步建立跨部门的市场监管综合执法机构。严格执行重大执法决定法制审核、行政执法人员持证上岗和资格管理制度，实行执法全过程记录。完善执法程序，明确操作流程，推行行政裁量权基准制度，全面落实行政执法责任制和责任追究制度。坚决排除对执法活动的干预，禁止野蛮、粗暴执法，切实做到严格、规范、公正、文明执法。严格执行市政府工作规则，推行行政复议委员会制度，按照法定程序和规章制度办事，推进政府工作的制度化、规范化。

　　（四）依法强化权力制约。加强公务员队伍建设，继续巩固和扩大党的群众路线教育实践活动成果，严格执行党的政治纪律、财经纪律和组织纪律。按照“谁主管、谁负责”的原则，认真落实党风廉政建设主体责任。实行公共资金、国有资产、国有资源和领导干部履行经济责任情况审计全覆盖，保障审计机关依法独立行使审计监督权，加快形成科学有效的权力运行制约和监督体系。严格落实“八项规定”和“约法三章”，坚决反对“四风”，严控“三公”经费和一般性开支，模范遵守《廉政准则》，严肃查处为政不廉行为，着力打造为民务实清廉政府。

　　各位代表，目标凝聚人心，实干铸就辉煌！让我们紧密团结在以习近平同志为总书记的党中央周围，在省委、省政府和市委的正确领导下，保持定力，真抓实干，为加快做大九江步伐、奋力开创“双核”发展新征程作出新的更大贡献！
